# Supplementary material for: Genome-wide screen in human plasma identifies multifaceted complement evasion of Pseudomonas aeruginosa
Source: PLoS Pathog. 2023 Jan 25;19(1):e1011023. doi: 10.1371/journal.ppat.1011023 (PMC9901815; doi:10.1371/journal.ppat.1011023)
Supplement: S1 Fig — Survival kinetics of IHMA87 wild-type strain (same data as presented in Fig 2B) and of Tn::Psrg in plasma over 6h incubation measured by CFU counting (n = 5). (DOCX) [file ppat.1011023.s001.docx]

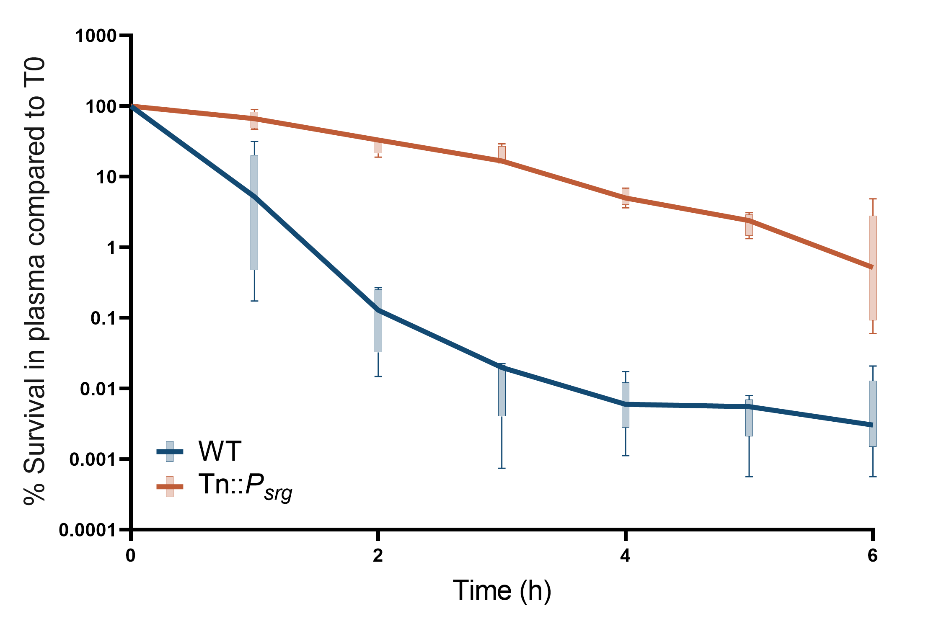


**S1 Fig. Overexpression of *srg* operon leads to a population tolerant to plasma killing.**Survival kinetics of IHMA87 wild-type strain (dame data as presented in Fig. 2B) and of Tn::*P_srg_* in plasma over 6h incubation measured by CFU counting (n=5).
