# Supplementary material for: Genome-wide screen in human plasma identifies multifaceted complement evasion of Pseudomonas aeruginosa
Source: PLoS Pathog. 2023 Jan 25;19(1):e1011023. doi: 10.1371/journal.ppat.1011023 (PMC9901815; doi:10.1371/journal.ppat.1011023)
Supplement: S2 Fig — A. The survival of IHMA87 WT, ΔbioB, ΔpurD and ΔpurDΔbioB was estimated by CFU counting and the median of all independent experiments is represented by the histogram. Statistical analysis was performed and p-value <0.05 or 0.01 are indicated with ‘*’ and ‘**’, respectively. B. Transmission electron microscopy images of IHMA87ΔpurDΔbioB after 1h-incubation in plasma. Scale bar = 500 nm. (DOCX) [file ppat.1011023.s002.docx]

**
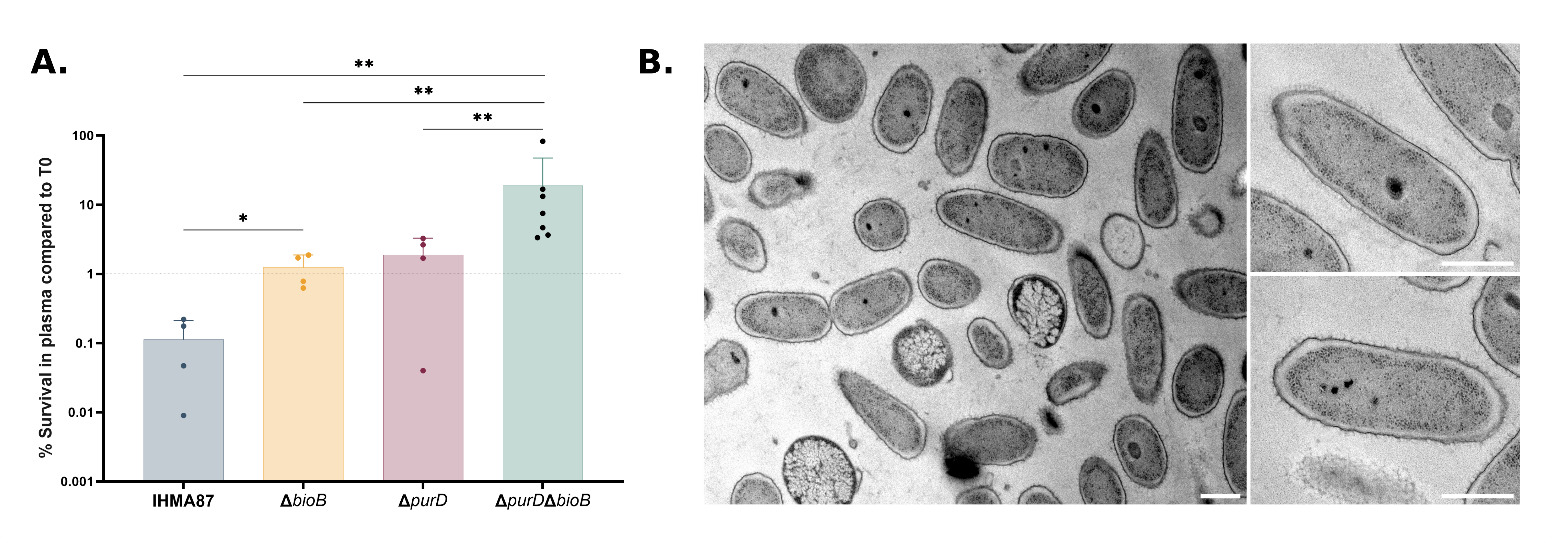
**

**S2 Fig. Depletion in biotin and purine increase bacterial survival to plasma independently from one another. A.** The survival of IHMA87 WT, Δ*bioB,* Δ*purD* and Δ*purD*Δ*bioB* was estimated by CFU counting and the median of all independent experiments is represented by the histogram. Statistical analysis was performed and p-value <0.05 or 0.01 are indicated with ‘*’ and ‘**’, respectively. **B.** Transmission electron microscopy images of IHMA87Δ*purD*Δ*bioB* after 1h-incubation in plasma. Scale bar = 500 nm.
