# Supplementary material for: Genome-wide screen in human plasma identifies multifaceted complement evasion of Pseudomonas aeruginosa
Source: PLoS Pathog. 2023 Jan 25;19(1):e1011023. doi: 10.1371/journal.ppat.1011023 (PMC9901815; doi:10.1371/journal.ppat.1011023)
Supplement: S3 Fig — Measurement of ATP levels in different plasma pools. ATP was not detected in LB. n = 3. (DOCX) [file ppat.1011023.s003.docx]

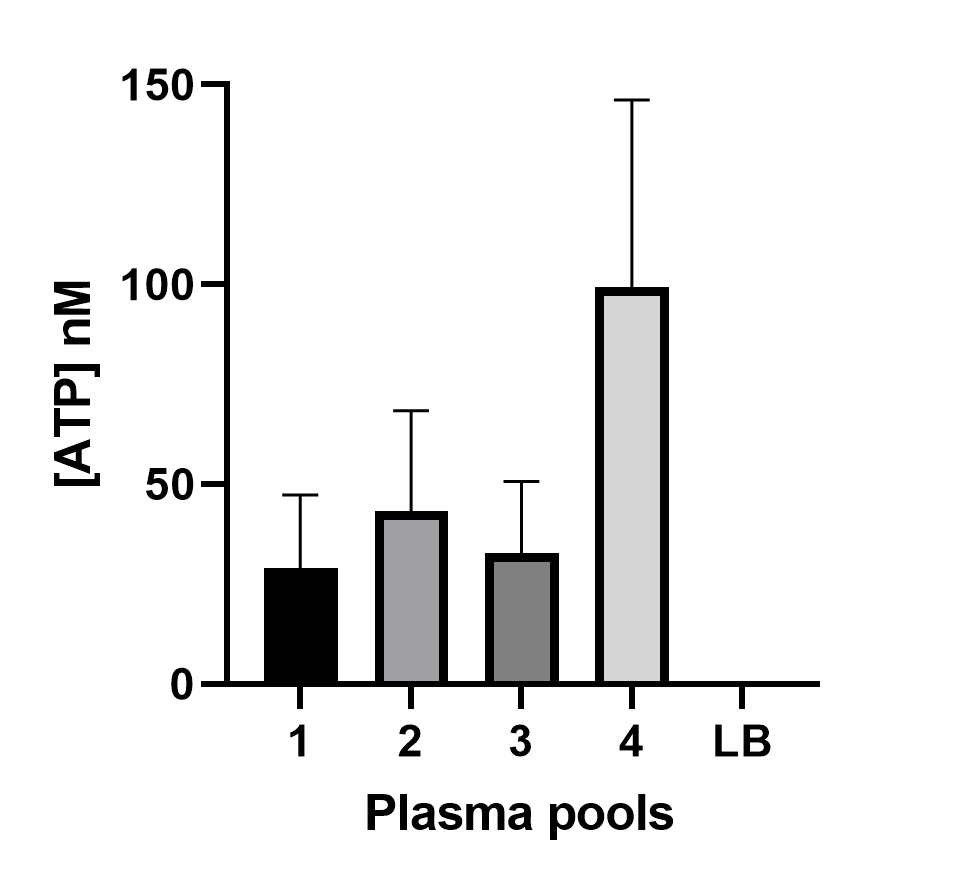


**S3 Fig. ATP concentration in LB and plasma pools.** Measurement of ATP levels in different plasma pools. ATP was not detected in LB. n=3.
