# Supplementary material for: Genome-wide screen in human plasma identifies multifaceted complement evasion of Pseudomonas aeruginosa
Source: PLoS Pathog. 2023 Jan 25;19(1):e1011023. doi: 10.1371/journal.ppat.1011023 (PMC9901815; doi:10.1371/journal.ppat.1011023)
Supplement: S4 Fig — A. Transmission electron microscopy images of ΔbioB after growth in LB (left) or 1h-incubation in human plasma (right). Arrows show small-sized granules. B. Transmission electron microscopy images of two BSI isolates PaG1 and PaG7 after growth in LB (left) or 1h-incubation in human plasma (right). Scale bar = 500 nm. (DOCX) [file ppat.1011023.s004.docx]

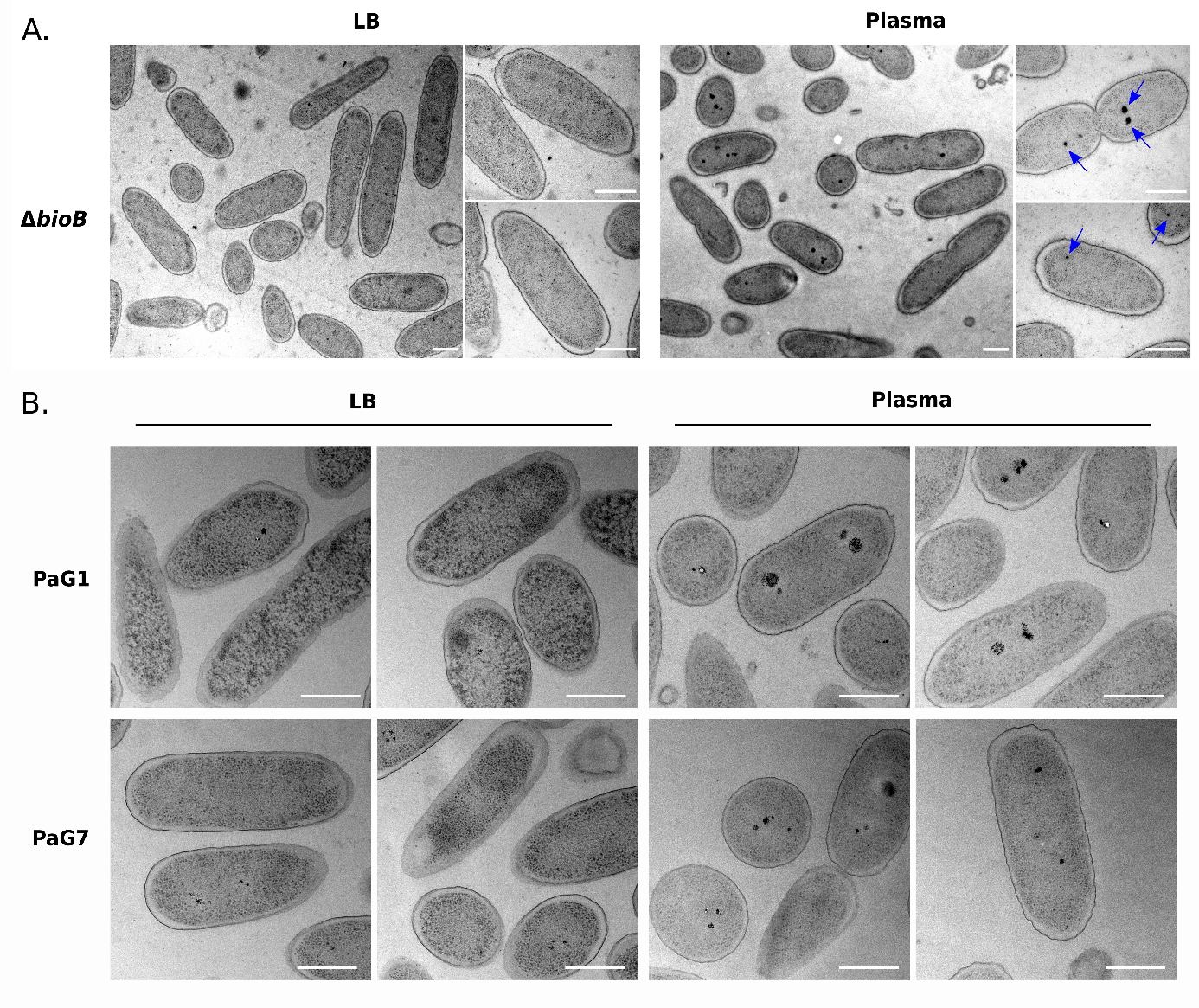


**S4 Fig. Incubation in plasma triggers polyphosphate granules formation. A.** Transmission electron microscopy images of Δ*bioB* after growth in LB (left) or 1h-incubation in human plasma (right). Arrows show small-sized granules. **B.** Transmission electron microscopy images of two BSI isolates PaG1 and PaG7 after growth in LB (left) or 1h-incubation in human plasma (right). Scale bar = 500 nm.
