# Supplementary material for: Genome-wide screen in human plasma identifies multifaceted complement evasion of Pseudomonas aeruginosa
Source: PLoS Pathog. 2023 Jan 25;19(1):e1011023. doi: 10.1371/journal.ppat.1011023 (PMC9901815; doi:10.1371/journal.ppat.1011023)
Supplement: S5 Fig — Transmission electron microscopy images of IHMA87 after 1h-incubation in heat-inactivated plasma (HIP, left) or in native human plasma (right). Scale bar = 500 nm. (DOCX) [file ppat.1011023.s005.docx]

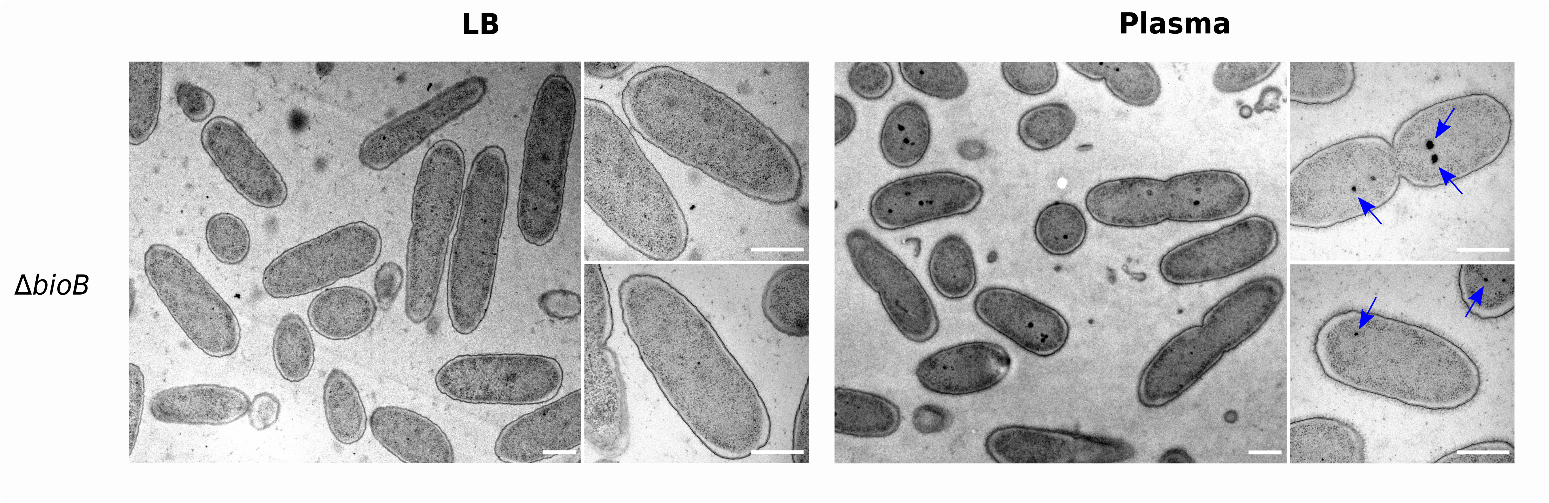


**S5 Fig. Heat inactivated plasma triggers the formation of polyP granules.** Transmission electron microscopy images of IHMA87 after 1h-incubation in heat-inactivated plasma (HIP, left) or in native human plasma (right). Scale bar = 500 nm.
