# Supplementary material for: Genome-wide screen in human plasma identifies multifaceted complement evasion of Pseudomonas aeruginosa
Source: PLoS Pathog. 2023 Jan 25;19(1):e1011023. doi: 10.1371/journal.ppat.1011023 (PMC9901815; doi:10.1371/journal.ppat.1011023)
Supplement: S3 Table — (DOCX) [file ppat.1011023.s008.docx]

**S3 Table. Bacterial strains and plasmids**

| **Bacteria** | **Features** | **Reference/origin** |
| --- | --- | --- |
| ***Pseudomonas aeruginosa*** |  |  |
| IHMA879472/AZPAE15042 | Urinary isolate, plasma sensitive | IHMA* collection [1–3] |
| IHMA87Δ*bioB* | IHMA87 with *bioB* deletion | This work |
| IHMA87Δ*purD* | IHMA87 with *purD* deletion | This work |
| IHMA87Δ*bioB*Δ*purD* | Double mutant | This work |
| IHMA87Δa*lgD* | IHMA87 with *algD* deletion | This work |
| IHMA87Δ*01134* | IHMA87 with *01134* deletion | This work |
| IHMA87Δ*ppk1* | IHMA87 with ppk1 deletion | This work |
| IHMA87Δ*ppk2* | IHMA87 with ppk2 deletion | This work |
| IHMA87Δ*ppk1*Δ*ppk2* | Double mutant | This work |
|  |  |  |
| IHMA87 Tn::*P_srg_* | IHMA87 with mariner transposon inserted in the promoter of *srg* operon (Gm^R^) | This work |
| IHMA87 Tn::*P_srg_* Δ*srgABC* | IHMA87 Tn::*P_srg_* with *srgABC* deletion (Gm^R^) | This work |
| IHMA87 Tn::*P_srg_* Δ*srgA* | IHMA87 Tn::*P_srg_* with *srgA* deletion (Gm^R^) | This work |
| IHMA87 Tn::*P_srg_* Δ*srgB* | IHMA87 Tn::*P_srg_* with *srgB* deletion (Gm^R^) | This work |
| IHMA87 Tn::*P_srg_* Δ*srgC* | IHMA87 Tn::*P_srg_* with *srgC* deletion (Gm^R^) | This work |
| IHMA87 Tn::*P_srg_* Δ*srgBC* | IHMA87 Tn::*P_srg_* with *srgBC* deletion (Gm^R^) | This work |
| IHMA87 Tn::*P_srg_* Δ*algD* | IHMA87 Tn::*P_srg_* with *algD* deletion (Gm^R^) | This work |
| IHMA87 Tn::*P_srg_* Δ*01134* | IHMA87 Tn::*P_srg_* with *01134* deletion (Gm^R^) | This work |
| IHMA87 *ladS*::Tn | IHMA87 with mariner transposon inserted in *ladS* gene (Gm^R^) | This work |
| IHMA87 *bioA*::Tn | IHMA87 with mariner transposon inserted in *bioA* gene (Gm^R^) | This work |
| IHMA87 *bioB*::Tn | IHMA87 with mariner transposon inserted in *bioB* gene (Gm^R^) | This work |
| IHMA87 *mucE*::Tn | IHMA87 with mariner transposon inserted in *mucE* gene (Gm^R^) | This work |
| IHMA87 *mucA*::Tn | IHMA87 with mariner transposon inserted in *mucA* gene (Gm^R^) | This work |
| IHMA87 *mucD*::Tn | IHMA87 with mariner transposon inserted in *mucD* gene (Gm^R^) | This work |
| IHMA87 *pprB*::Tn | IHMA87 with mariner transposon inserted in *pprB* gene (Gm^R^) | This work |
| IHMA87 *mucB*::Tn | IHMA87 with mariner transposon inserted in *mucB* gene (Gm^R^) | This work |
| PaG1 | Bloodstream isolate, O8, infected organ | [2] |
| PaG7 | Bloodstream isolate, O1, contaminated catheter | [2] |
| ***Escherichia coli*** |  |  |
| DH5α | Laboratory strain | Lab collection |
| TOP10 | Cloning strain | Invitrogen |
| **Plasmids** |  |  |
| pBTK24 | Plasmid with Himar-1 mariner transposon and C9 transposase (Amp^R^, Gm^R^) | [4] |
| pRK600 | Helper plasmid with conjugative properties (Cm^R^) | [5] |
| pEXG2 | Allelic exchange vector (Gm^R^), *sacB* | [6] |
| pEX18Tc | Allelic exchange vector (Tc^R^), *sacB* | [7] |
| pEX100T | Allelic exchange vector (Cb^R^), *sacB* | [8] |
| pminiCTX-*pX2*-GFP | pminiCTX harboring the *gfp* gene under the control of a constitutive promoter (*attP* site, Tc^R^) | [9] |
| pminiCTX-*Psrg*-*lacZ* | pminiCTX-*lacZ* harboring the srg promoter fused to *lacZ* reporter gene (*attP* site, Tc^R^) | This work |
| pEXG2-mut-*bioB* | pEXG2 carrying DNA fragment for *bioB* deletion obtained by SLIC (Gm^R^) | This work |
| pEXG2-mut-*purD* | pEXG2 carrying DNA fragment for *purD* deletion obtained by SLIC (Gm^R^) | This work |
| pUC57-mut-*srgABC* | pUC57 carrying the synthetic DNA fragment for *srgABC* deletion in Tn::*P_srg_* (Genewiz) | This work |
| pEX18Tc-mut-*srgABC* | pEX18Tc carrying DNA fragment for srgABC deletion in Tn::*P_srg_* mutant obtained by SLIC (Tc^R^) | This work |
| *pEX18Tc-mut-01134* | pEX18Tc carrying DNA fragment for IHMA87_01134 deletion obtained by SLIC (Tc^R^) | This work |
| pEX100T-mut-*srgA* | pEX100T carrying DNA fragment for *srgA* deletion obtained by SLIC (Cb^R^) | This work |
| pEX100T-mut-*srgB* | pEX100T carrying DNA fragment for *srgB* deletion obtained by SLIC (Cb^R^) | This work |
| pEX100T-mut-*srgC* | pEX100T carrying DNA fragment for *srgC* deletion obtained by SLIC (Cb^R^) | This work |
| pEX100T-mut-*srgAB* | pEX100T carrying DNA fragment for *srgAB* deletion obtained by SLIC (Cb^R^) | This work |
| pEX100T-mut-*srgBC* | pEX100T carrying DNA fragment for *srgBC* deletion obtained by SLIC (Cb^R^) | This work |
| pEX100T-mut-*algD* | pEX100T carrying DNA fragment for *algD* deletion obtained by SLIC (Cb^R^) | This work |
| pEXG2-mut-ppk1 | pEXG2 carrying DNA fragment for *ppk1* deletion obtained by SLIC (Gm^R^) | This work |
| pEXG2-mut-ppk2 | pEXG2 carrying DNA fragment for *ppk2* deletion obtained by SLIC (Gm^R^) | This work |

* International Health Management Association, USA

**References**

1. Kos VN, Deraspe M, McLaughlin RE, Whiteaker JD, Roy PH, Alm RA, et al. The resistome of Pseudomonas aeruginosa in relationship to phenotypic susceptibility. Antimicrob Agents Chemother. 2015 Jan;59(1):427–36.

2. Pont S, Fraikin N, Caspar Y, Van Melderen L, Attree I, Cretin F. Bacterial behavior in human blood reveals complement evaders with some persister-like features. PLoS Pathog. 2020 Dec;16(12):e1008893.

3. Trouillon J, Sentausa E, Ragno M, Robert-Genthon M, Lory S, Attree I, et al. Species-specific recruitment of transcription factors dictates toxin expression. Nucleic Acids Res. 2020 Mar 18;48(5):2388–400.

4. Kulasekara HD, Ventre I, Kulasekara BR, Lazdunski A, Filloux A, Lory S. A novel two-component system controls the expression of Pseudomonas aeruginosa fimbrial cup genes. Mol Microbiol. 2005;55(2):368–80.

5. Kessler B, de Lorenzo V, Timmis KN. A general system to integratelacZ fusions into the chromosomes of gram-negative eubacteria: regulation of thePm promoter of theTOL plasmid studied with all controlling elements in monocopy. Mol Gen Genet MGG. 1992 May;233(1–2):293–301.

6. Rietsch A, Vallet-Gely I, Dove SL, Mekalanos JJ. ExsE, a secreted regulator of type III secretion genes in Pseudomonas aeruginosa. Proc Natl Acad Sci. 2005 May 31;102(22):8006–11.

7. Li S, Li X, Zhao H, Cai B. Physiological role of the novel salicylaldehyde dehydrogenase NahV in mineralization of naphthalene by Pseudomonas putida ND6. Microbiol Res. 2011 Dec 20;166(8):643–53.

8. Schweizer HP, Hoang TT. An improved system for gene replacement and xylE fusion analysis in Pseudomonas aeruginosa. Gene. 1995 Jan 1;158(1):15–22.

9. Thibault J, Faudry E, Ebel C, Attree I, Elsen S. Anti-activator ExsD forms a 1:1 complex with ExsA to inhibit transcription of type III secretion operons. J Biol Chem. 2009 Jun 5;284(23):15762–70.
