# Supplementary material for: Genome-wide screen in human plasma identifies multifaceted complement evasion of Pseudomonas aeruginosa
Source: PLoS Pathog. 2023 Jan 25;19(1):e1011023. doi: 10.1371/journal.ppat.1011023 (PMC9901815; doi:10.1371/journal.ppat.1011023)
Supplement: S4 Table — (DOCX) [file ppat.1011023.s009.docx]

**S4 Table. Oligonucleotides used for PCRs**

| **Primers** | **Sequence (5’-3’)** |  |
| --- | --- | --- |
| pEXG2-mut-*bioB*-sF1 | **GGTCGACTCTAGAGGATCCCC**GTTCGGACCCCTCGAACATCG | *bioB* deletion |
| pEXG2-mut-*bioB*-sR1 | GGTGGCGACGGCTGCGGAT | *bioB* deletion |
| pEXG2-mut-*bioB*-sF2 | **GCGCATCCGCAGCCGTCGCCACC**CAGCTGTTCTATAACGCCGCCT | *bioB* deletion |
| pEXG2-mut-*bioB*-sR2 | **ACCGAATTCGAGCTCGAGCCC**CTTGCCGACCAGCGCGGTGA | *bioB* deletion |
| pEXG2-mut-*purD*-sF1 | **GGTCGACTCTAGAGGATCCCC**CAGGAGATCCACGACCTGATCT | *purD* deletion |
| pEXG2-mut-*purD*-sR1 | ACCGCCGCTGCCGATGATGA | *purD* deletion |
| pEXG2-mut-*purD*-sF2 | **TACTCATCATCGGCAGCGGCGGT**GAGCGCGGCGAGTCCTGAC | *purD* deletion |
| pEXG2-mut-*purD*-sR2 | **ACCGAATTCGAGCTCGAGCCC**TCGCGGACCAGCTGGCCGT | *purD* deletion |
| pEXG2-mut-ppk1-sF1 | **GGTCGACTCTAGAGGATCCCC**AGTGAAGATGGCCGAAACGATC | *ppk1* deletion |
| pEXG2-mut-ppk1-sR1 | **TCA**GCTCTTGTTCACCAGCAGCGG | *ppk1* deletion |
| pEXG2-mut-ppk1-sF2 | **CTGCTGGTGAACAAGAGCTGA**CAGTACCCGCCCTTCACCCC | *ppk1* deletion |
| pEXG2-mut-ppk1-sR2 | **ACCGAATTCGAGCTCGAGCCC**AGCGTCTTGCCCATCCCGATC | *ppk1* deletion |
| pEXG2-mut-ppk2-bis-sF1 | **GGTCGACTCTAGAGGATCCCC**GCGAGCCTGCCGGCGAACTA | *ppk2* deletion |
| pEXG2-mut-ppk2-bis-sR1 | **TCA**CTTGAACAGCAGGATGCCGCT | *ppk2* deletion |
| pEXG2-mut-ppk2-bis-sF2 | **GGCATCCTGCTGTTCAAGTGA**GACGAGAAGGTCTACGCCGAG | *ppk2* deletion |
| pEXG2-mut-ppk2-sR2 | **ACCGAATTCGAGCTCGAGCCC**GCCAGAAGGTCACCGGCCTG | *ppk2* deletion |
| pEX18Tc-mut-*srgABC*-sF1 | **GTCGACTCTAGAGGATCCC**CGGATCCCCGACATTCGGCTA | Transfer DNA fragment from pUC57 to pEX18Tc for *srgABC* deletion in Tn::*P_srg_* |
| pEX18Tc-mut-*srgABC*-sR1 | **CGAATTCGAGCTCGGTACCC**AAGCTTTGATCTACGTGCAAGC |  |
| F0–mut-*srgABC* | GCCTCGCCGACCTCTACA | PCR verification for *srgA, srgB, srgC* and *srgABC* deletion |
| RO-Tn::*P_srg_*-mut-*srgABC* | GCTTGCTGCCTTCGACCAAG | PCR verification for *srgA, srgB, srgC* and *srgABC* deletion |
| pEX18Tc-mut-01134-sF1 | **GTCGACTCTAGAGGATCCCC**CACCTCGGTGTCCACGCTGC | IHMA87_01134 deletion |
| pEX18Tc-mut-01134-sR1 | CGACGAGCATCGACTTGTTCAC | IHMA87_01134 deletion |
| pEX18Tc-mut-01134-sF2 | **TGAACAAGTCGATGCTCGTC**GACAAGAACGGTCGGCTGGT | IHMA87_01134 deletion |
| pEX18Tc-mut-01134-sR2 | **CGAATTCGAGCTCGGTACCC**ATGTTCCGCAGCCTGGTCGG | IHMA87_01134 deletion |
| F0-mut-01134 | CTGCTGTACGTCGGCTTCCG | PCR verification for IHMA87_01134 deletion |
| R0-mut-01134 | CTGGATGGCACCACCAACTTC | PCR verification for IHMA87_01134 deletion |
| pEX100T-mut-*srgA*-sF1 | **ACCCTGTTATCCCTACCC**GTGATCTACGTGCAAGCAGA | *srgA* and *srgAB* deletion in Tn::*P_srg_* |
| pEX100T-mut-*srgA*-sR1 | CAGGTTGCGTGAGCTGCTCA | *srgA* and *srgAB* deletion in Tn::*P_srg_* |
| pEX100T-mut-*srgA*-sF2 | **TGAGCAGCTCACGCAACCTGTGA**CCTGGACATTCTGACGAGGTA | *srgA* deletion in Tn::*P_srg_* |
| pEX100T-mut-*srgA*-sR2 | **GGATAACAGGGTAATCCC**CACCAGCGCAAGCAGCAGC | *srgA* deletion in Tn::*P_srg_* |
| pEX100T-mut-*srgB*-sF1 | **ACCCTGTTATCCCTACCC**ATGGAGGTGAACATGAGCAGC | *srgB* and *srgBC* deletion in Tn::*P_srg_* |
| pEX100T-mut-*srgB*-sR1 | AGCCAGAATCCACATGTTTC | *srgB* and *srgBC* deletion in Tn::*P_srg_* |
| pEX100T-mut-*srgB*-sF2 | **GAAACATGTGGATTCTGGCTTGA**ACGGAAGCTCGGCGCCACT | *srgB* deletion in Tn::*P_srg_* |
| pEX100T-mut-*srgB*-sR2 | **GGATAACAGGGTAATCCC**CGTGTTGACTCACGTCGGAC | *srgB* deletion in Tn::*P_srg_* |
| pEX100T-mut-*srgC*-sF1 | **ACCCTGTTATCCCTACCC**CGACGAACAGGCCTGGACAT | *srgC* deletion in Tn::*P_srg_* |
| pEX100T-mut-*srgC*-sR1 | AAGAATGACGGCGGATCGTGA | *srgC* deletion in Tn::*P_srg_* |
| pEX100T-mut-*srgC*-sF2 | **TCACGATCCGCCGTCATTCTTTGA**GATCCCAGCCTGGACTGATC | *srgC* deletion in Tn::*P_srg_* |
| pEX100T-mut-*srgC*-sR2 | **GGATAACAGGGTAATCCCC**CACTCGAAGCCGACATTCG | *srgC* deletion in Tn::*P_srg_* |
| pEX100T-mut-*srgAB*-sF2 | **TGAGCAGCTCACGCAACCTGTGA**ACGGAAGCTCGGCGCCACT | *srgAB* deletion in Tn::*P_srg_* |
| pEX100T-mut-*srgAB*-sR2 | **GGATAACAGGGTAATCCC**CGTGTTGACTCACGTCGGAC | *srgAB* deletion in Tn::*P_srg_* |
| pEX100T-mut-*srgBC*-sF2 | **GAAACATGTGGATTCTGGCTTGA**GATCCCAGCCTGGACTGATC | *srgBC* deletion in Tn::*P_srg_* |
| pEX100T-mut-*srgBC*-sR2 | **GGATAACAGGGTAATCCCC**CACTCGAAGCCGACATTCG | *srgBC* deletion in Tn::*P_srg_* |
| pEX100T-mut-*algD*-sF1 | **ACCCTGTTATCCCTACCC**CGGAAACGCCATCAAGTTGGTA | *algD* deletion |
| pEX100T-mut-*algD*-sR1 | GCCAGCACATACCGCACCGA | *algD* deletion |
| pEX100T-mut-*algD*-sF2 | **TCGGTGCGGTATGTGCTGGC**CAGGCCGAGGGCATCTGCT | *algD* deletion |
| pEX100T-mut-*algD*-sR2 | **GGATAACAGGGTAATCCC**GCTGTCGATGGCTTCGCGGA | *algD* deletion |
| F0-mut-*algD* | CTCGTGGCGAATAGGCCTAC | PCR verification for *algD* deletion |
| R0-mut-*algD* | GCGAAGCGCAGCTTGTGCC | PCR verification for *algD* deletion |
| **Primers for Tn-seq** |  |  |
| Short adaptor | TACCACGACCA-NH2 |  |
| Long adaptor | GTGACTGGAGTTCAGACGTGTGCTCTTCCGATCTGGTCGTGGTAT |  |
| PCR1 Tn-specific | CACAGGAAACAGGACTCTAGAGG |  |
| PCR2 adaptor complementary | GTGACTGGAGTTCAGACGTGTG |  |
| P5+ Illumina | AATGATACGGCGACCACCGAGATCTACACTCTTTCCCTACACGACGCTCTTCCGATCTCTAGAGACCGGGGACTTATCAGC |  |
| P7-index | CAAGCAGAAGACGGCATACGAGAT**CGTGAT** |  |
