# Supplementary material for: Genome-wide screen in human plasma identifies multifaceted complement evasion of Pseudomonas aeruginosa
Source: PLoS Pathog. 2023 Jan 25;19(1):e1011023. doi: 10.1371/journal.ppat.1011023 (PMC9901815; doi:10.1371/journal.ppat.1011023)
Supplement: Attachment — Submitted filename: PLOS Pathogens_Janet-Maitre et al_responses to reviewers.pdf [file ppat.1011023.s010.pdf]

PPATHOGENS-D-22-01021

Molecular features underlying *Pseudomonas aeruginosa* persistence in human plasma

---

Dear Dr Guillard,

Thank you for taking time to evaluate our work submitted to *PLOS Pathogens*. We appreciate the input -yours and that of the reviewers-, and we have revised this version accordingly. We revised the text and added new data and figures, which include the data with survival rates of double *purD-bioA* mutant (Fig. S2) and survival rates and electron microscopy of deletion mutants in *ppk1*, *ppk2* and of the double *ppk1-ppk2* mutant (Fig. 6).
As suggested by the reviewers, we clarified several parts of the manuscript and underlined the multifactorial mechanism of *Pseudomonas aeruginosa* evasion. In line, the results of the screen are now summarized in the last schema (Fig. 8). In agreement with reviewers' global critics and suggestions, we propose to change the title of our manuscript to "Genome-wide screen in human plasma identifies multifaceted complement evasion of *Pseudomonas aeruginosa*".
Please, find enclosed the revised manuscript as well as a point-by-point response to your comments, as well as to the reviewers' comments. We hope that this revision will make our manuscript suitable for *PLOS Pathogens*.

<u>Editor comments</u>

…….While this paper represents a large body of work, it appears somehow built on new descriptions of three genes, which conferred to P. aeruginosa ability to persist in human plasma, but with no clear explanation whether they interplayed in the whole mechanism.

As now emphasized throughout the manuscript, the identified factors contribute independently to bacterial evasion in plasma. There are several data supporting our conclusion: first, the externally added ATP and biotin do not *trans*-complement the *bio* and *pur* mutants, respectively. Moreover, in the revised version of the manuscript we present the phenotype of the double *purD-bioB* mutant which shows even better survival than the individual mutants (please see Fig.S2). The same is the case for SrgA which acts independently. We now prepared the Fig. 8 that schematically shows the different pathways, including here-identified, that lead to complement evasion.

….In this paper, we understand that human plasma is a low ATP environment, which is a stress signal for P. aeruginosa that in turn forms polyP granules as phosphate storage to compensate for low intracellular concentration of ATP. As a consequence, P. aeruginosa resists MAC killing thanks to a membrane that is more resistant or that repairs faster.

Indeed, our conclusion is that the plasma is **a stress signal** and a trigger for polyP formation. However, we cannot conclude that the plasma is a low ATP environment. We have measured the ATP concentration in plasma, and compared it to LB. We found that the overall ATP concentration between 15 to 45 nM (Fig. S3) in plasma is higher than in LB (undetected), suggesting that plasma is not a low ATP environment for the bacteria. The Δ*purD* mutant is under stress (probably due to low intracellular ATP pool, or purine depletion) even in LB which leads to the formation of polyP granules. In this mutant, the low intracellular ATP mimics what happens in plasma. Both events lead to the membranes that are more resistant to MAC.

As raised by the referees #1 and #3, the description of the mechanism deployed by P. aeruginosa to persist in human plasma lacks a clear <u>global picture</u> allowing to connect all the pathways you have identified. Indeed, it is hard to eventually link the biotin biosynthesis pathway to the purin biosynthesis pathway to the srgABC operon. A final figure to sum up the

mechanism of persistence of P. aeruginosa in human blood would have been very helpful and would strengthen your manuscript.

As suggested by the reviewers, we have included the final Figure in the manuscript.

The major issues….

The first major issue that I would like to reach to your attention is the mechanism that triggers phosphate storage granules in P. aeruginosa in response to ATP starvation. It is not very clear if the plasma is poor in ATP and therefore the bacterium is found to have low intracellular ATP or if the plasma is a stress signal that is detected by P. aeruginosa, which responds by modulating the intracellular synthesis of ATP. Indeed, the plasma supplementations with ATP restoring sensitivity seem in favor of the 1st hypothesis. However, on page 17 it is written "bacteria could use regulatory mechanisms to modulate purin and biotin synthesis…" which seems to explain that low intracellular levels of ATP may result from a decrease in biosynthesis in response to human plasma.

Everything goes in favor of the second hypothesis. As mentioned previously, the stress encountered in plasma is the main trigger for polyP granule formation. The low intracellular ATP concentration and/or purine starvation in *purD* mutant is a stress for the bacteria which also in turn triggers polyP formation (Jung et al., 2012).

Consequently, the text on page 17 has been modified. We believe indeed, that there are several possibilities in which the bacteria can face different ATP concentrations; one is variable ATP concentrations in human serum (Human metabolome database; Gorman et al., 2007; Mempin et al., 2013)

In other words, does low intracellular level of ATP result from uptake from a poor ATP environment or of decreased synthesis in response to plasma?

Again, the important aspect here is not the ATP *per se*, but the formation of polyP granules that is enhanced in a *pur* mutant or triggered by plasma in wild-type strains. Indeed, our new results show that mutants in *ppk* genes (*ppk*1 and *ppk*2) encoding polyphosphate kinases are unable to persist in the plasma. Therefore, polyP are critical in the fight with the complement system (please see new Fig. 6). As shown in Fig. S3, ATP levels in plasma are not a trigger for polyphosphate granules formation. Indeed, in LB, which has no detected ATP, bacteria are devoid of polyP granules.

The second major issue raised by reviewer #1 and #3 is the lack of clarity when linking membrane resistance to MAC with the low intracellular level of ATP to fully describe the mechanism of persistence of P. aeruginosa in plasma. How polyP granules and ATP starvation lead to MAC resistant membranes is very difficult to understand. Moreover the description of the Srg operon turns out to be only described here without any role in the ATP starvation response described previously. - How to connect ATP formation from polyP granules by Ppk and Srg in resistance to MAC killing?

As mentioned previously, there is no direct link identified so far between *bio*, *pur* and *srg.* However, both ATP starvation in *pur* mutants or incubation in plasma lead to polyP formation and subsequently to increase survival in plasma. Our new results show that the inactivation of *ppk1* and *ppk2* leads to a 30-fold drop in median bacterial survival (see Fig. 6C).

The three pathways lead to increased plasma resistance due to inefficient MAC activity. The reason for impaired MAC activity may be of multiple origin, the bacterial envelope modification being the most plausible.

- In BSI due to a WT bacterium, how the purin/biotin synthesis pathway as well as Srg are modulated since the evader phenotype is reversible and no genetic mutations occur? Eventually, the Srg story seems very promising but very preliminary. Unless you can connect the three pathways all in one mechanism explaining the plasma persistence deployed by P.

aeruginosa in human blood, I guess your manuscript would benefit by focusing on one of the mutants. The ΔpurD is likely to be more straightforward in the attempt to describe the response upon ATP starvation (plasma stress signal, signal transduction and polyP granules, MAC killing membrane resistance).

As mentioned previously, our screen identified several novel independent pathways, all leading to increased bacterial resistance to MAC-killing. One of the key findings is that the evasion is highly multifactorial. This is now summarized in an additional Figure. The presented results are opening a new perspective to this work, and the authors do not wish to drop out the Srg story.

I would like to raise your attention on some minor (because not contradicting the main message of your paper) issues. 1. Could you specify if your findings are also true for human blood and not only human plasma? Indeed, if it is fully understandable that demonstration of persistence should have been made in plasma, it is important to link your results to that which occurs in BSI.

We did not continue our experiments with human blood, because of two reasons: 1. there is too much variability between individuals to make a clear conclusion, and 2. we cannot pool blood from different individuals.

2. Could you explain in the manuscript why you worked with ΔbioB and not mutants with clean deletions in other pur genes?

3. Could you explain in the manuscript why you worked with ΔpurD and not mutants with clean deletions in other bio genes?

To validate Tn-seq, we systematically constructed the « clean » deletions in top-hit genes from the screen. For the *pur* pathway, we selected the second hit, for *bio*, the third hit which is not in the operon with other *bio* genes. This has been now explained in the text to read: “we designed a deletion mutant of *purD* (Δ*purD*), given its non-operonic structure” (page 13, lines 262-263). We also constructed a double mutant *purD-bioB* (Fig. S2)

4. Page 17 you stated that “although incapable of growth, ΔbioB and ΔpurD mutants survived and persisted in HIP”. Could you clarify in the manuscript if these mutants are incapable of growth in vitro or in HIP? In the first case, it implies that you should explain how you could grow such mutants before incubating them within HPI. In the second case, it implies that you should explain if there is killing or growth inhibition in HPI?

We are sorry for this confusion. The mutants grow in LB, although slower, and similarly to the wt strains, grow poorly in HIP, but persist over a long period. We deleted this statement from the manuscript so as not to bring confusion to the reader.

5. Page 19, second paragraph, could you be more specific when using “these bacteria”? For instance, it is difficult to understand if “these bacteria” written just after “In evaders” are the mutants mentioned at the beginning of the paragraph or P. aeruginosa that evaded human plasma bactericidal activity in your experiments with WT or clinical strains.

Thank you. This has been corrected.

Reviewer #1 comments

We would like to thank the reviewer for constructive comments and suggestions. We have modified the main text in order to take into account the main critics and added additional experiments. We believe that our new results contribute to general conclusions of our work.

…Although no mechanisms was completely elucidated (which was stated as the goal in the first sentence in the discussion) …

We agree with the reviewer. The screen identified novel factors that allow bacteria to evade CS. Elucidation of all molecular mechanisms was not the scope of the present manuscript. This sentence has been modified, and we propose to change the title of the manuscript.

Potential weaknesses include that the MS in its current form is more descriptive - it would benefit from more focus on one or two pathways.

We kept the three candidates as we believe that one key take-home message from our work is the multifactorial interaction between *P. aeruginosa* and the complement system. However, to go further in connecting polyP with persistence in plasma, we created additional mutants in the genes *ppk1* and *ppk2* (encoding polyphosphate kinases) and evaluated their survival in plasma, which directly showed the role of polyP in persistence. Also, we added a final scheme summarizing all our data.

Part II – Major Issues: Key Experiments Required for Acceptance

The authors wrote "The aim of this study was to elucidate the mechanisms deployed by P. aeruginosa to persist in human plasma", however, they have not followed through with this goal.

Our work highlights the complexity of bacterial persistence in human plasma. It paves the way for future studies which will investigate those novel evasion mechanisms at molecular level. We have changed the text accordingly (page 17, lines 374-376). Please see our answer above.

They have identified several pathways that appear to affect persistence in plasma, however, no direct mechanism is provided. In fact, the reviewer is wondering how a ΔpurD strain, which is characterized by low ATP levels due to impaired purine biosynthesis, is able to produce such substantial amounts of polyP, which PPK can only generate from ATP.

Bacterial intracellular ATP concentrations are in the millimolar range (1-5mM, Mempin et al., 2013). Therefore, a 20% reduction still leaves the bacterium with a high intracellular concentration which may be sufficient for the generation of polyP. Moreover, Jung et al., 2012 showed in *E. coli* that a purine mutant produced more polyP.

Part III – Minor Issues: Editorial and Data Presentation Modifications

1) The manuscript is written for an audience with direct expertise in the field. It would likely attract more readers if more background information was provided, e.g. it would benefit from a more thorough introduction into the complement system.

According to the reviewer's suggestion, we have now explained better the biological pathways important for the global understanding of our work. Notably, we introduce key proteins on the complement system in the Introduction (see page 5, lines 86-94).

2) Page 7: "However, the screen did confirm the importance of LPS and more specifically OSA....". This was hard to find for the reviewer, the authors may want to help the reader which transposon mutants they are referring to.

We are sorry if our data was not sufficiently explained. We now indicated in the text to which hit we referred to (P_{wzz1}) and described the role of *wzz1* gene product (page 8, lines 177-179).

3)Page 8: "This effect is of interest as Psl improved bacterial survival.....". It is also unclear hear, how retS/ladS and Psl relate to each other.

We detailed in the text the link between RetS/LadS and Psl (please see page 7-8, lines 158-164).

4) Page 11: survival increased ~20-fold according to Figure 2C. It would also be less confusing if the authors only use srgABC throughout the text as they do in the figures. They introduce the new names only in the 4th sub-panel.

We do agree with the suggestion and have modified the text accordingly. We introduced the *srg* name before, on page 10, line 199.

In our hands, even with a pool of plasma, the killing assays are highly variable; we show that Tn::P_{srg} has improved survival in plasma which can reach 100 fold.

5) Page 11: The authors claim that all thee srg genes are highly conserved in P. aeruginosa, which is not too surprising. Are there homologs in other bacterial species and, if so, what are those?

Given the high genetic diversity within *P. aeruginosa* species, and the small size of the core genome (665 genes, Freschi et al., 2019), it is rather surprising to see three genes encoding small proteins of unknown function being part of the core genome (as small proteins are usually less conserved). SrgC shows some homology with proteins outside of *P. aeruginosa* (with a poor coverage, <45%, representing one transmembrane helix), however, the two others do not, which is common for small proteins.

6) Fig 2 E and F are not described in the text.

Thank you for noting this mistake. The part of text was removed along the process of editing. We included the part of the text that was missing (please refer to page 11, lines 212-225).

7) The authors decided to construct a ΔpurD strain, why not a purM or purF mutants, as those genes had much higher Tn insertion frequencies?

The gene *purF* is in an operon with two other genes whose expression could have been affected by a *purF* deletion through mRNA destabilization, therefore we have decided to mutate *purD* which has the second highest Log_2(FC).

8) Fig 4C: Any explanation why ATP and/or biotin addition doesn't affect the WT? Does the cell only take these compounds up when in desperate need? The reviewer also wonders why the replicate numbers differ throughout the experiments.

The reviewer raises some interesting points. The absence of an effect on the WT could be due to a threshold under which biotin concentration is critical. In the case of *purD* mutant, the depletion in ATP triggers the stress and higher production of polyP, a key factor of the CS evasion according to our work. Please see new results with *ppk* mutants (Fig. 6).

For replicate numbers: in our experience, because of the high variability of results, three independent experiments were not sufficient to obtain statistically significant results, even if the tendency could be seen. That is why we performed in the most cases additional replicates.

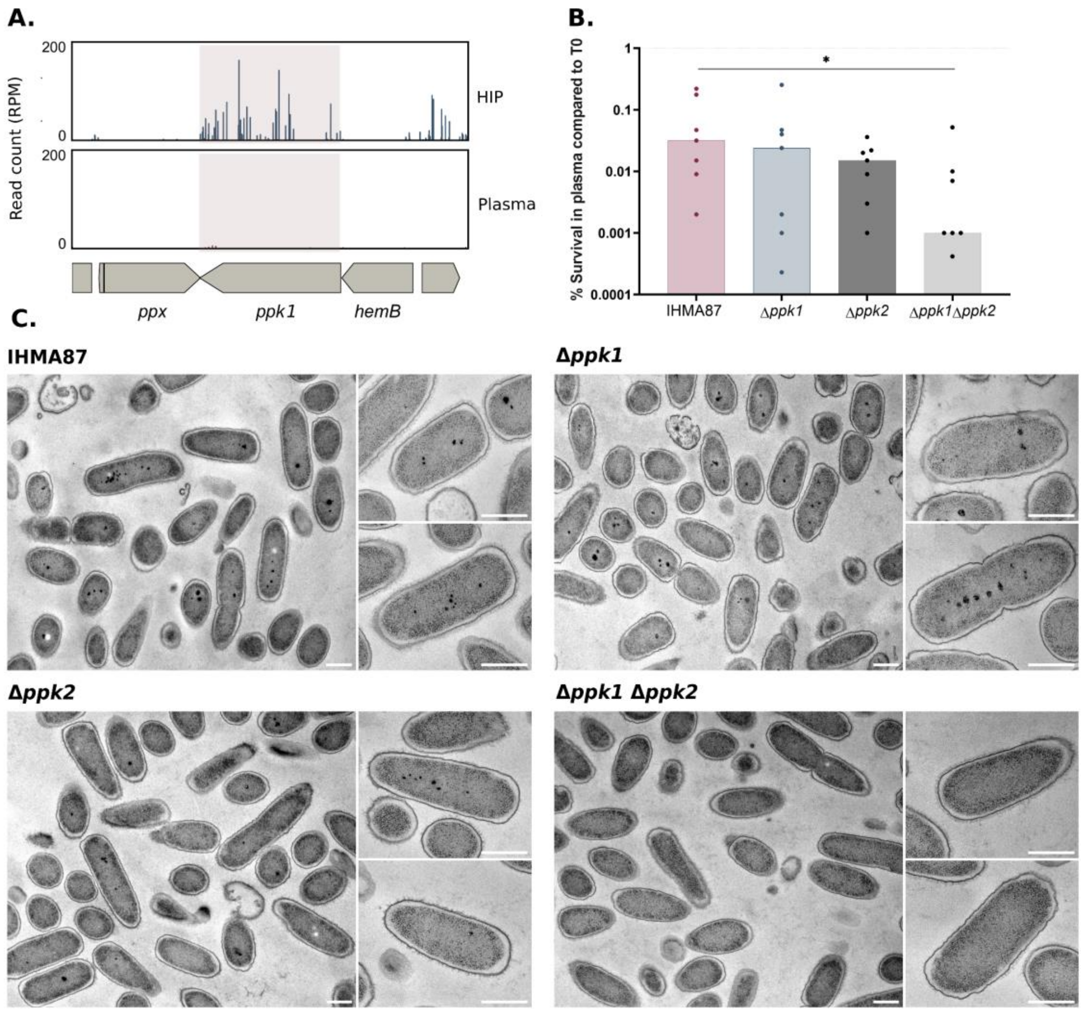

Figure 6. Polyphosphates are critical for evaders' survival in plasma. A. Zoom in on Tn-seq profiles of *ppk1* gene with 2000 bp upstream and downstream, showing normalized numbers of reads in input (HIP) and output (plasma) samples. **B.** Survival of IHMA87 wild-type strain, individual Δ*ppk1*, Δ*ppk2* or double Δ*ppk1*Δ*ppk2* deletion mutants following incubation for 3 h in plasma. Survival rates were calculated based on CFU measurements and the median of all independent experiments is represented by the histogram. Statistical analysis was performed and *p*-value <0.05 is indicated with '*'. **C.** Transmission electron microscopy images of IHMA87, Δ*ppk1*, Δ*ppk2* or Δ*ppk1*Δ*ppk2* after 1h-incubation in human plasma. Scale bar = 500 nm.

Reviewer #2 comments

… Unfortunately, the novelty is somewhat lacking because it is already common knowledge that PPK is relevant to PA virulence and survival in host from prior studies. It is thus not surprising to see in this paper that ΔPPK strains were not able to survive in plasma. Overall, I don't think there is strong ground to conclude that polyP is important to cope with plasma-related stress.

Our work is the first report of a link between polyP and the CS in *P. aeruginosa*. We now have direct proof that polyP are important, as the double *ppk1-ppk2* mutants do not survive CS-mediated killing (please see new Figure 6). Indeed, it has been shown that the polyP are important for *P. aeruginosa* motility, biofilm formation and virulence in animal models (Rashid et al., 2017), but molecular mechanisms have not been described.

Here is another example of insufficient data for drawing their conclusions. Their study only confirmed that ATP levels could influence PA tolerance in one mutant (ΔpurD). While in the other mutant (Δbio), it seems that ATP levels are not important?

We do not imply that ATP is the only factor of bacterial resilience to CS. In addition, in *purD* mutant, the increased survival in plasma is rather due to higher polyP production. We believe that in the new version of the manuscript we underlined the multifactorial mechanisms of *P. aeruginosa* CS evasion. Our data suggest diverse mechanisms which are now summarized in the last Figure 8.

In "Analysis of isolated mutants" section they discuss but do not show their results?

The results for this section are described in the paragraph 'analysis of Tn mutants isolated during the screen' and the results are shown in the Fig. 1C.

Part III – Minor Issues: Editorial and Data Presentation Modifications

Abstract:

human plasma and blood – plasma is a component of blood

Modified in the text.

Author Summary:

biotin to significantly influence bacterial capacity to deal with - biotin significantly influence bacterial capacity to deal with

The text has been modified.

Page 5:

between 0.01 and 0.1% of evaders - between 0.01% and 0.1% of evaders

Modified.

Page 7:

Random colony-picking failed to identify any LPS-related mutants - How many/percentage of colonies were picked up

10 mutants were picked up. The aim of isolating those mutants was to confirm the Tn-seq results and have isolated mutants in hands.

Two isolated displayed hyper-mucoid phenotypes – Two isolated mutants?

Indeed, two of the mutants that were isolated had a mucoid phenotype and were affected in either *mucA* or *mucD*.

Page 8:

Interestingly, bioinformatics analysis of the sequencing data revealed significant enrichment of transposon insertions in retS, which codes for a LadS antagonist (Table 1). This effect is of interest as Psl improved bacterial survival of a mucoid strain in serum (Jones and Wozniak, 2017). - First mention of Psl, need to elaborate. What's the relationship between retS and Psl?

We have now added what is known about the relationship between RetS/LadS and exopolysaccharides Psl (please see page 7, lines 158-164).

Our data thus suggest that either type IVb pili or CupE fimbriae could be also involved in P. aeruginosa plasma resilience, but the mechanisms involved need to be explored further. - which data, which figure?

A transposon mutant in the promoter of *pprB*, which positively regulates Type IV pili and CupE fimbriae has an increased survival in plasma. This data is shown on Fig.1B and described on page 8, lines 169-174.

Page 10: Together, those results confirm the role of LPS, alginates, and Psl in resistance to the CS, validating the Tn-seq approach - What results? How do they confirm that LPS, alginates and Psl are resistance to CS? and how to validate Tn-seq?

The results described in the chapter (p 10) came from the bioinformatics analysis of our Tn-seq datasets. They were then confirmed by plasma killing assay of individual mutants in the case of *mucA*, *mucD, mucE* (alginates) and ladS (Psl), as presented in Fig. 1B. As we mentioned, both alginates and polysaccharides Psl were already described in the literature as affecting complement-mediated killing and therefore they validate our screen.

By the way, they might have forgotten to cite references in some sentences which is confusing since it is hard to know where the information is coming from.

We have added references for clarity.

Reviewer #3 comments

Authors raise the question of the mechanisms involved in persistence of P. aeruginosa in human plasma but without exposing a full explanation on discovered mechanisms. Some hypothesis would need more experimental approaches.

To go further concerning the link between polyP and tolerance, we designed deletion mutants in both polyphosphate kinases *ppk1* and *ppk2* and assessed survival of mutants in plasma (Fig. 6). In addition, we designed a double mutant Δ*purD*Δ*bioB* and assessed its survival in plasma to further demonstrate that those two evasion mechanisms are independent (Fig. S2).

Part II – Major Issues: Key Experiments Required for Acceptance

The emergence of so called "evaders" could result of mucoid overproduction, Psl production or type IV pili involvement. These data were already described in literature.

We disagree with the reviewer for both points: first, the evaders, as defined in our first publication Pont *et al.*, 2020, were never described before in the literature and second, we do not conclude that mucoid overproduction, Psl or T4P are involved in evader phenotype. In our work, the hits in *muc* genes and in *ladS* just validate the screen. New factors found in this screen improve survival in plasma in an alginate-, Psl- and type 4 pili - independent manner.

In Janet-Maitre et al., described novel mechanisms: biotin and purine metabolisms and a new called serum resistance gene. The ***authors underlined the involvement of multiple and diverse mechanisms for human plasma persistence****. However, even if the identification of*

these mechanisms is important, the link between the identified genes and persistence phenotype stays hypothetical. The investigation of serum factors triggering this reaction will be interesting.

We thank this reviewer for underlying our data as "***multiple and diverse mechanisms for human plasma persistence".*** However, we do not understand the question, which reaction the reviewer is referring to. We showed in our previous publication that the complement system in plasma is essential for the elimination or sensitive population, therefore evaders persist to complement activity. Finally, we agree that "*The investigation of serum factors triggering this reaction will be interesting",* but this investigation is out of scope of the present work.

Experiments on BSI isolates are needed to confirm the involvement of identified pathways. I am afraid that the mutants obtained in the "in vitro" conditions are not reflecting the in vivo conditions.

We used two BSI isolates to examine polyP granules and observed that polyP granules are produced in BSI strains upon plasma incubation.

If we understood the concern of the reviewer correctly, we can underline that the evaders are genetically identical (without the mutation in the genome) to the rest of the bacterial population. Evaders are also reversible and represent a tiny population. So, the work here aimed to explore potential pathways modulating survival in plasma. Indeed, the mutations identified here are not expected to arise specifically in the context of a bacteremia.

It is difficult to follow the different parts of the publication on purine, biotine and srgA pathways, as they have no link to each other. Authors should focus on one of these pathways to try to decipher the mechanisms involved with the use of double mutants for example. The focus could be on the formation of PolyP granules.

We believe that one important take home message from the paper is the multifactorial origin of evaders and that a combination of all those factors will result in the population of evaders. As suggested by the reviewers, we modified the text to make it clearer. We also studied the connection between the *purD* and *bioB* pathways by creating a double mutant and assessing its survival to human plasma (please see additional results described in Fig S2). We also included a final scheme (Fig. 8) which summarizes our data to help the reader go through those different pathways.

Concerning purine pathways, the finality will be the reservoir of energy under PolyP granules, and the decrease of ATP. However, the mechanisms involved are not clear.

Indeed, the mechanism by which polyP interfere with MAC-dependent killing is beyond the scope of this paper. However, we proposed a few hypotheses including that polyP formation modify outer membrane constituents (lipids, proteins and lipoglycans), as proposed for *Campylobacter jejuni* (Pina-Mimbela et al., 2015). In this case, it could directly interfere with MAC insertion into the outer membrane. In another study, in eukaryotes, polyP were found to destabilize C5b,6 complexes therefore affecting MAC formation (Conway et al., 2019).

About EDX experiments, the variations on osmium peak are puzzling between granules and cytoplasm but also between WT and mutant strain. It is definitely higher in the mutant and many other peaks that have appeared in the granule area were not labeled (fig 5B). Is this attributable to higher lipid environment (e.g. for osmium) or increased count number on the area (e.g. as seen for copper signal that is intended to come from the grid). How many total counts were acquired for each condition? Even if one can agree with the authors that there is a presence of phosphorus, it is not reasonable to conclude on the quantity without better explanations on the standardization process for EDX data acquisition.

We would like to stress out that the goal of the EDX experiment was to verify and identify the granule content, and not in any case to quantify the different components. The zoom-in image clearly shows that the selected areas contain much more phosphorus (P) in granule than in cytoplasm in both, wild-type and the *purD* mutant. The same seems to be true for oxygen (O), but we did not zoom-in that part of the image. We would like not to discuss at this point other components identified which seem to be in a background.

Authors wrote:

1. "In the context of higher tolerance/persistence to antibiotics, it has been proposed that the ATP-depleted conditions could decrease overall protein synthesis, and thus availability of antibiotic targets (Shan et al., 2017). Similarly, slow growth per se, whatever the restrictions causing it, can trigger a persistent state (Pontes and Groisman, 2019). Although incapable of growth, ΔbioB and ΔpurD mutants survived and persisted in HIP for more than 24 hours."

--> But other mutants with slow growth or low ATP were not identified? It seems only purine and biotin dependant?

We agree with the reviewer. The screen can identify other mutants impacted in the growth, however, in Tn-seq we cannot assess growth of the mutants therefore cannot conclude about the "slow growth" phenotypes. However, purine and biotin biosynthetic pathways were redundant with several hits within each of those pathways.
In our screen, we did not isolate any other mutants in the ATP production, showing the specificity of the purine pathway.

2. "Admittedly, mutations in the purine and biotin pathways are unlikely to occur in vivo, but bacteria could use regulatory mechanisms to modulate purine and biotin synthesis, to enter into an antibiotic- or complement system-tolerant state"

-->The other point is the link with what really happens in reality, in vivo. Why these mutants could not be isolated in vivo? Was the phenotype of real plasma evaders already studied? Could it possible to create mutant with induction/control of purine or biotin expression to confirm this hypothesis? Could it be possible to evaluate these gene expression in evaders isolated from BSI?

As for the antibiotic persistence, plasma persistence does not involve any mutation as it is a fully reversible phenotype. This is a reason why we propose that in a stochastic or regulated manner, individual bacteria in a population could regulate purine, biotin or polyP production and lead to transient tolerance to complement-mediated killing. However, as the phenotype is transient, evaders arise from the same clonal population. Through this study, we use Tn-seq as a tool to overtake those limitations and identify mechanisms which modulate bacterial resilience to plasma. Indeed, it would be interesting to find a way to isolate the evaders and perform RNA-seq to study whether their transcriptome differs from the bulk population. Although this is important, the project would require several years of investigation and is therefore out of scope of this paper.

3. "The number of plasma evaders present following incubation in plasma varies between experiments, strains and the plasma used ((Pont et al., 2020) and this work). Therefore, it is possible that the initial incubation which is considered poorly nutritive and stressful for bacteria may stochastically trigger the emergence of evaders." --> Could subcultures in plasma be possible to avoid the impact of initial incubation.

The proportion of evaders is constant, independently of the inoculum, as described in Pont et al. In line with this hypothesis, as opposed to persisters to antibiotics, a stationary phase bacterial population does not give rise to higher evaders population (Pont et al, 2020). In

addition, preincubation of bacteria in inactivated plasma for 2h prior to incubation in native plasma, didn't lead to a higher proportion of evaders.

However, our study here shows that incubation in heat-inactivated plasma is sufficient to trigger polyP granules formation, suggesting an adaptive bacterial response to plasma conditions.

4. “In evaders, in contrast, the outer membrane seems to have been effectively damaged by the MAC, suggesting that these bacteria have a more resistant envelope or can repair envelope damage more rapidly.”

--> What are the mechanisms involved in MAC (only membrane impact?)?

--> Comparison of evaders and mutants have to be done. Maybe by controlling /testing the fluidity of the membrane after the MAC experiment.

The reviewer raises some very interesting questions. Many aspects of MAC biology remained to be understood. This could be subject of future investigations; however, it is beyond the scope of this paper. In addition, measuring membrane fluidity in evaders will certainly be tricky given the reversibility of their phenotype.

Part III – Minor Issues: Editorial and Data Presentation Modifications

1. In result part:“The size and number of the electron dense granules increased upon incubation of ΔpurD in human plasma (Fig. 5A, red arrows)”

-->The size depends on the site of the cut. It is therefore necessary to moderate this observation.

For the reviewer (but could be added as supplementary data in the manuscript), we show here the low magnification images (1200x) in which bacteria appear in random orientations, probably representing the whole population. We can clearly see that the *purD* mutant has generally bigger granules (independently of the cut).

IHMA87 Plasma

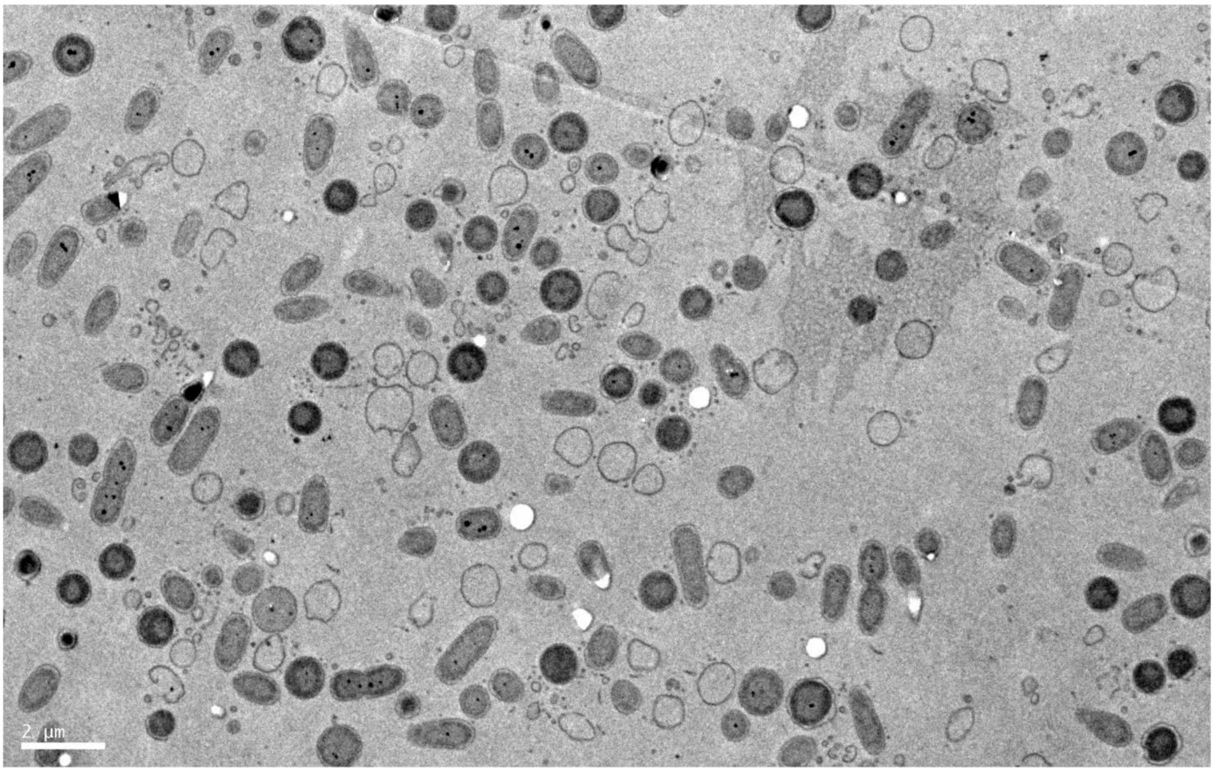

Δ*purD* Plasma

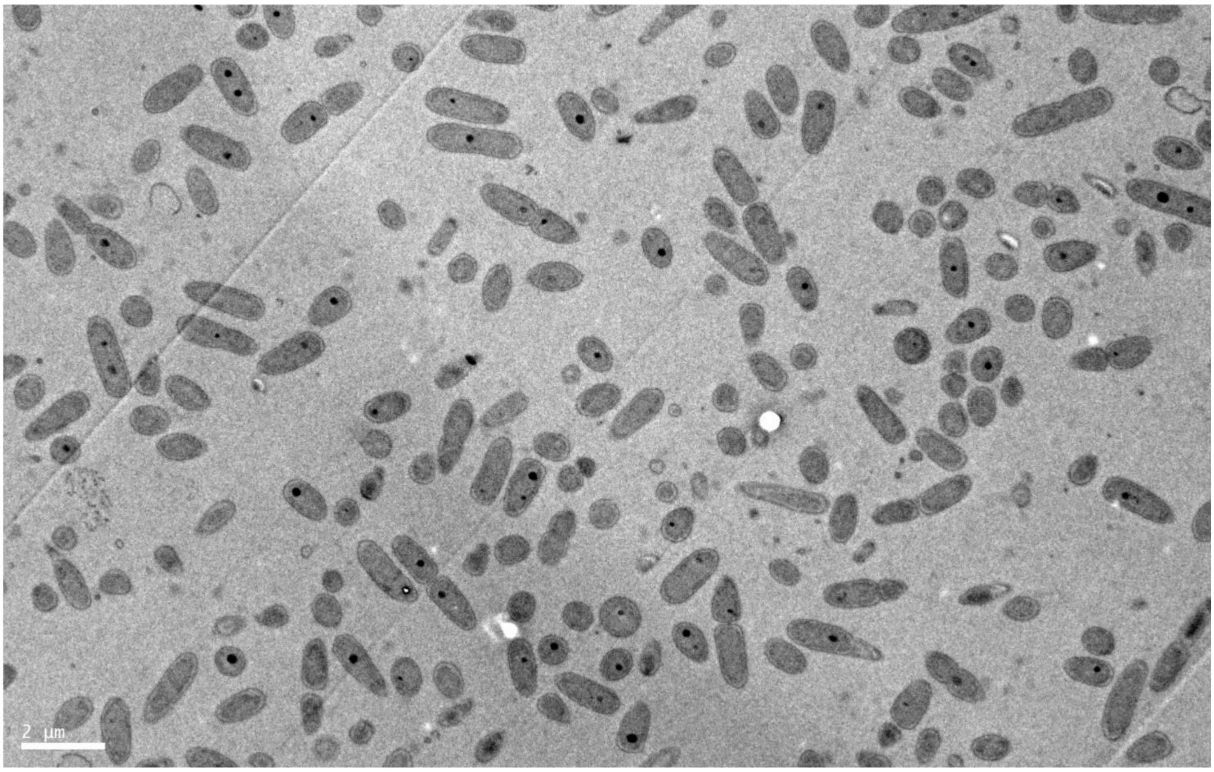

2. Screen or screening? Not sure of the term used. Both could be used.

3. I will propose a scheme for conclusion to clarify some points. For the sake of clarity, we included a final figure, summarizing our findings (please see Fig. 8).
